# Importance of Individualized Pressure Settings in Mechanical Insufflation-Exsufflation for Lung Volume Recruitment: A Case Report

**DOI:** 10.7759/cureus.84211

**Published:** 2025-05-16

**Authors:** Keiichi Funo, Toshio Fukutake, Ryoko Takeuchi, Yoshihiro Uzawa

**Affiliations:** 1 Rehabilitation, Kameda Medical Center, Kamogawa, JPN; 2 Neurology, Kameda Medical Center, Kamogawa, JPN

**Keywords:** amyotrophic lateral sclerosis, lung volume recruitment, mechanical insufflation-exsufflation, neuromuscular disease, respiratory care

## Abstract

Lung volume recruitment (LVR) has been proposed as a treatment to maintain respiratory health in patients with neuromuscular diseases who frequently develop restrictive ventilatory impairment due to muscle weakness. LVR applies noninvasive mechanical pressure techniques to maintain and improve pulmonary and chest wall compliance and to preserve vital capacity. Various methods of LVR have been developed, which can be classified into two types: the stacked-breath method and the single-breath method. Mechanical insufflation-exsufflation (MI-E) is one approach categorized under the single-breath method. Although the clinical use of pressure settings in MI-E varies, inspiratory pressure levels around 40 cmH₂O are sometimes applied in practice. However, such settings may result in patient discomfort and raise safety concerns. Given the limited clinical guidance available, it may be more appropriate to determine individualized settings based on each patient’s impairment level, pulmonary mechanics, and tolerance. This case report describes such an approach to LVR using the single-breath method with MI-E in a patient with amyotrophic lateral sclerosis (ALS). To determine the optimal inspiratory pressure, three parameters were assessed at each pressure level: expiratory volume, subjective perception of lung expansion, and immediate subjective effects following inspiration. As the patient reported discomfort at 30 cmH₂O, the final inspiratory pressure was set at 25 cmH₂O. This level of inspiratory assistance led to improvements in vocal loudness and alleviated breathlessness during speech. These positive effects contributed to the patient's acceptance of the intervention and its continued use after discharge to home care. This case highlights the importance of tailoring LVR settings to optimize effectiveness, patient comfort, and safety, based on pulmonary mechanics, bedside volume assessment, and patient-reported respiratory status.

## Introduction

Amyotrophic lateral sclerosis (ALS) is a progressive neurodegenerative disorder that affects both upper and lower motor neurons, resulting in generalized motor dysfunction, respiratory muscle weakness, dysarthria, and dysphagia [1,2]. Respiratory insufficiency in ALS is characterized by a progressive decline in vital capacity, primarily due to diaphragmatic involvement, eventually leading to the need for ventilatory support [1]. Chronic hypoventilation, associated with an impaired ability to perform deep inspirations, contributes to reduced thoracic compliance and the development of restrictive ventilatory impairment. Lung volume recruitment (LVR) techniques are employed in individuals with neuromuscular diseases, including ALS, to preserve tidal volume and improve pulmonary and thoracic compliance [3,4]. LVR is a therapeutic intervention that transiently increases lung volume to maintain or enhance pulmonary compliance. Mechanical insufflation-exsufflation (MI-E), a noninvasive technique that simulates natural coughing by alternating positive and negative airway pressures, is one method commonly utilized for LVR. LVR strategies are generally classified into stacked-breath and single-breath methods [4]. The stacked-breath method includes glossopharyngeal breathing, manual hyperinflation using a self-inflating bag, and commercial devices such as the lung insufflation capacity (LIC) TRAINER2 [4-7], whereas the single-breath method comprises pressure-limited non-invasive ventilation (NIV), intermittent positive pressure breathing (IPPB), and MI-E [4,5]. These approaches aim to achieve either maximum insufflation capacity (MIC) or LIC, depending on whether glottic control is required [3-5]. Prior studies have demonstrated that achieving MIC or LIC may contribute to slowing the decline of forced vital capacity (FVC), which reflects not only respiratory muscle strength but also the flexibility of the lungs and chest wall, maintaining peak cough flow (PCF), a measure of cough strength, and improving pulmonary compliance in the short term [8-13].

Despite these potential benefits, the current evidence base supporting LVR remains limited, and its clinical application is often guided by expert consensus or extrapolated data rather than disease-specific research [4]. In particular, there is a lack of established guidance regarding optimal inspiratory pressure settings when applying MI-E in ALS. A previous review [14] proposed the use of inspiratory pressures of ≥40 cmH₂O, drawing upon studies conducted in non-neuromuscular populations, such as those involving acute respiratory distress syndrome (ARDS) and perioperative patients. As such, the generalizability of these recommendations to ALS remains questionable.

This case report presents an individual with ALS who underwent LVR using MI-E. Although initial pressure settings were informed by the prior literature, the patient experienced discomfort at 30 cmH₂O. The insufflation pressure was subsequently adjusted based on objective measures and the patient’s subjective response. This case underscores the importance of individualized pressure setting strategies in LVR using MI-E and proposes a pragmatic approach that integrates bedside assessment with patient tolerance.

## Case presentation

**Figure 1:**
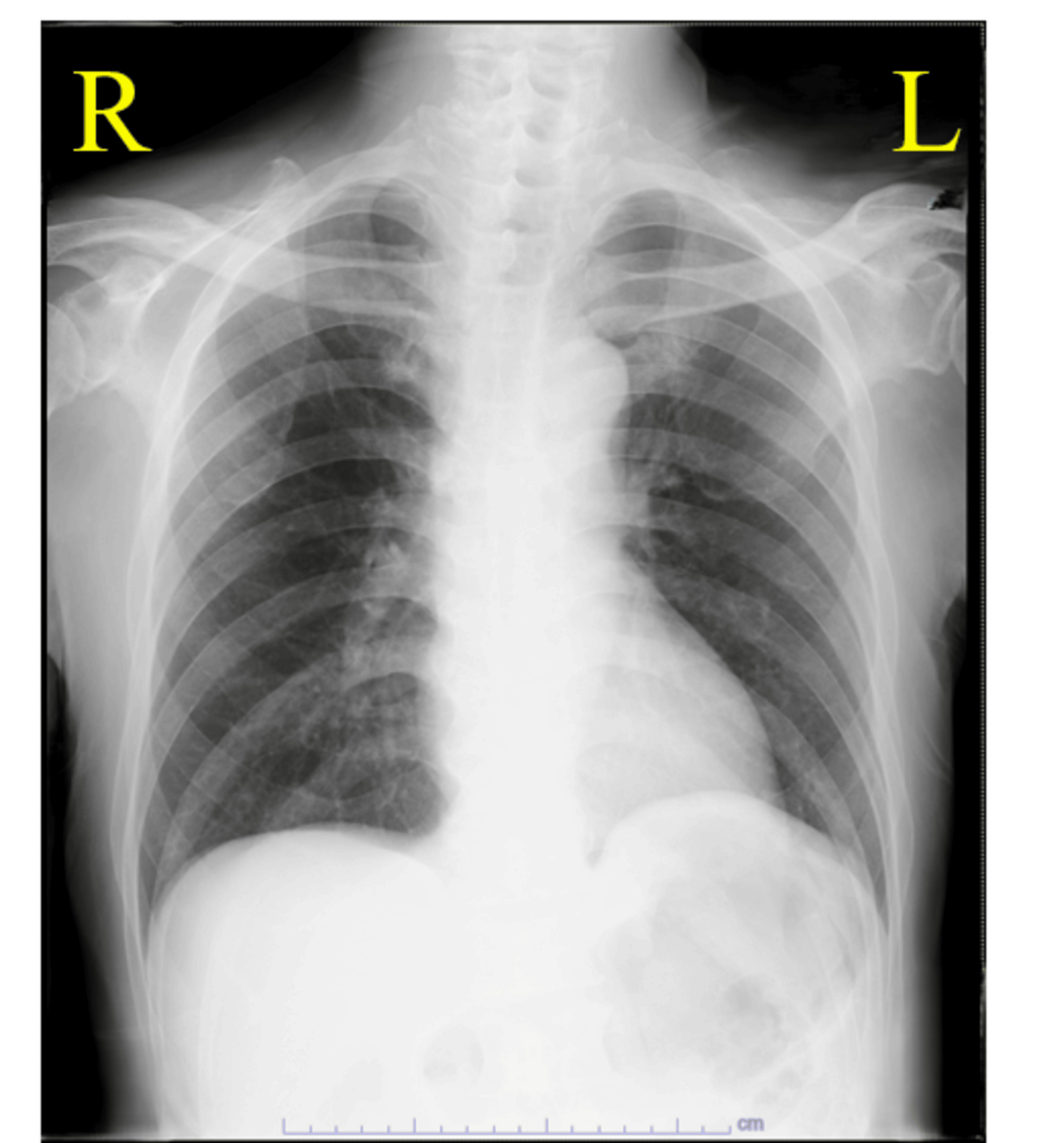
Chest X-ray image at admission

The patient was a male in his 60s diagnosed with ALS; his height was 160 cm, weight 55.3 kg, and BMI 21.6. He had no history of cardiovascular or pulmonary disease (Figure 1, Tables 1-2). He first noticed a decline in grip strength five years prior to admission and was diagnosed with ALS three years ago. The clinical presentation was consistent with a slowly progressive, upper limb-onset form of classical ALS, with bulbar and respiratory symptoms appearing later in the disease course. Two years ago, he underwent percutaneous endoscopic gastrostomy (PEG) and subsequently continued to receive home care. Although the patient had undergone PEG, he was still able to consume regular meals orally. Voluntary cough was weak, indicating reduced airway clearance, while a detailed assessment of pharyngeal and glottic closure function was not conducted.

**Table 1:** Laboratory data on admission This table summarizes the patient's key laboratory values at the time of admission. All results were within normal ranges, except for a mildly elevated C-reactive protein (CRP), which was not clinically significant. WBC = white blood cell count; SpO₂ = saturation of percutaneous oxygen; BUN = blood urea nitrogen; BNP = brain natriuretic peptide

Test	Value	Reference range
CRP	3.31 mg/dL	<0.3 mg/dL
WBC	7,000/μL	4,000-9,000/μL
SpO₂ (room air)	97%	≥96%
Albumin	3.6 g/dL	3.5-5.0 g/dL
BUN	11 mg/dL	8-20 mg/dL
Creatinine	0.41 mg/dL	0.6-1.2 mg/dL
Hemoglobin	14.3 g/dL	13.0-17.0 g/dL
BNP	5.2 pg/mL	<18.4 pg/mL

**Table 2:** Arterial blood gas measurements BE: base excess

Test	Value	Reference range
pH	7.41	7.35-7.45
PaCO₂	45.1	35-45mmHg
PaO₂	88.7	80-100mmHg
HCO₃	28.6	20-26 mmoL/L
BE (vt）	3.3	-3.3 to 2.3 mmoL/L

**Figure 2:**
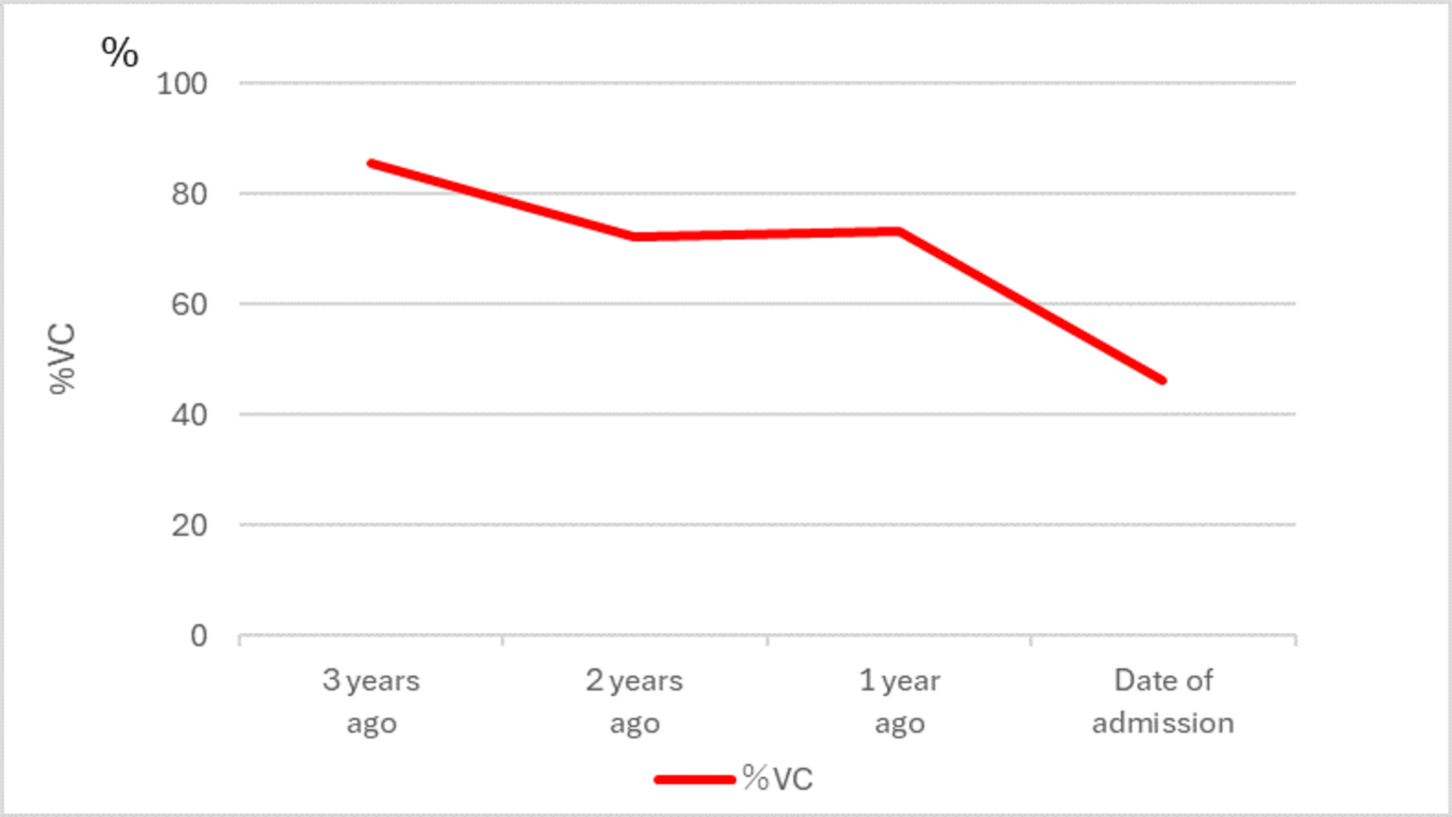
Progress of pulmonary function tests (progress of %VC) %VC = ％ vital capacity

At the time of admission, he exhibited reduced vocal volume, dyspnea during conversation, and dysphagia. In terms of activities of daily living (ADL), he required total assistance from family members at home. Figure 2 illustrates the progressive decline in his respiratory function over the preceding three years. Due to the anticipated progression of ALS, he was admitted for the initiation of non-invasive ventilation and assessment of LVR using an MI-E device.

Method

Comfort Cough II (Seoil Pacific Corp., Seoul) was utilized as the MI-E device. To determine the optimal inspiratory pressure for LVR, three key parameters were evaluated at each pressure setting: (1) expiratory volume, (2) subjective perception of lung expansion, and (3) immediate subjective effects following inspiration (such as vocal volume and dyspnea).

The MI-E had previously been set at an inspiratory pressure of +20 cmH₂O to support his cough and expectoration. The inspiratory pressure settings were increased in increments of 5 cmH₂O starting from +20 cmH₂O; inspiratory time was standardized at two seconds, consistent with the settings used for secretion clearance. The intervention was conducted with the patient seated in a reclining wheelchair, with the backrest tilted at approximately 30 degrees. LVR was performed using the MI-E device with an interface mask. The patient was instructed to actively inhale in synchrony with the device's insufflation phase. The expiratory volume was measured by temporarily disconnecting the MI-E circuit from the interface mask and attaching a spirometer directly to the mask. Measurements followed American Thoracic Society (ATS) standards, with the mean of three trials recorded. The patient’s subjective perception of lung expansion was assessed immediately after the intervention using a 10-point Numeric Rating Scale (NRS), where 0 indicated no perceived lung expansion and 10 indicated maximum perceived expansion. Additionally, physical therapists evaluated the immediate post-procedure effects based on subjective verbal responses regarding voice volume, ease of speech, and sensation of dyspnea.

Results

**Figure 3:**
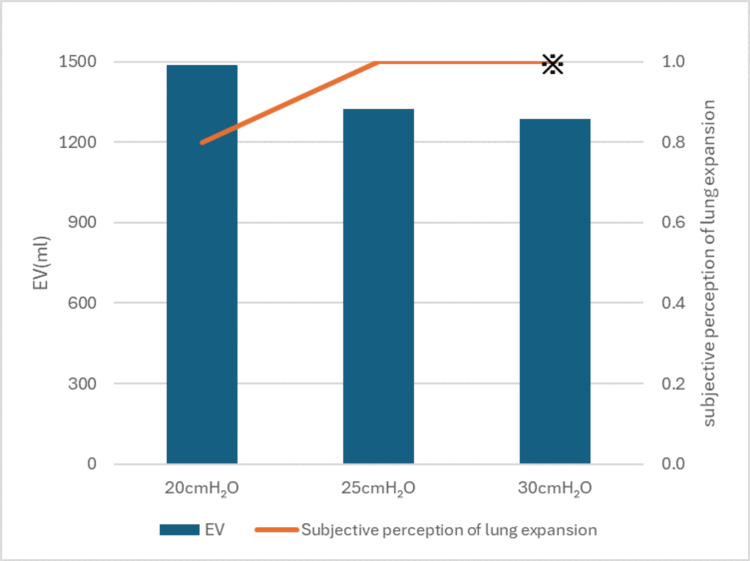
Expiratory volume and subjective perception of lung expansion EV = expiratory volume ^※^Although the subjective perception of lung expansion was rated as 1.0, the patient reported that excess air was being delivered.

The results of the measurements are presented in Figure 3. The expiratory volume was 1.48 L at an inspiratory pressure of +20 cmH₂O, 1.32 L at +25 cmH₂O, and 1.28 L at +30 cmH₂O. Subjective perception of lung expansion was 0.8 at +20 cmH₂O, and 1 at both +25 cmH₂O and +30 cmH₂O. However, during the evaluation at +30 cmH₂O, the patient reported feeling excess air was being delivered. Regarding the immediate post-inspiratory effects, an inspiratory pressure of +25 cmH₂O provided more immediate benefits than +20 cmH₂O, with improvements in vocal volume and a reduction in breathlessness during conversation. Based on these findings, the inspiratory pressure was set to +25 cmH₂O. The expiratory volume increased by approximately 160 mL when moving from +20 to +25 cmH₂O. However, no further increase was observed at +30 cmH₂O, suggesting a plateau in volume gain. Subjective ratings of lung expansion reached a maximum at +25 cmH₂O, and +30 cmH₂O was associated with discomfort. These findings indicate that +25 cmH₂O represented an optimal balance between volume recruitment and tolerability in this case.

## Discussion

Our case questions the applicability of a uniform inspiratory pressure threshold (e.g., 40 cmH₂O) across diverse clinical contexts. A previous review [4] underscored the limited evidence supporting the clinical efficacy of LVR, attributing the ongoing debate to a paucity of rigorous prospective studies. In light of this, a more cautious and individualized approach is warranted. The present case reinforces this perspective, illustrating the value of incorporating assessments of pulmonary mechanics, respiratory muscle performance, and patient-reported symptoms such as dyspnea and airway coordination. The following section discusses the rationale behind previously reported inspiratory pressure targets and the considerations informing our individualized adjustments.

A prior report [14] recommended pressures of ≥40 cmH₂O for MI-E, based on studies of alveolar recruitment in ARDS models [15] and re-expansion of atelectasis during general anesthesia [16]. However, these studies were conducted in contexts unrelated to ALS, such as animal models or patients without neuromuscular disease, and may not reflect the pathophysiology of ALS-related respiratory impairment. These discrepancies highlight the limitations of directly applying such targets to ALS patients and underscore the need for individualized adjustment. In our case, discomfort was reported even at 30 cmH₂O, prompting us to abandon the initially referenced target. De Troyer and Deisser hypothesized that the low pulmonary compliance seen in muscular dystrophy patients results from chronic atelectasis, which may not be readily reversible by short-term lung inflation [17]. Mechanisms underlying reduced pulmonary compliance in neuromuscular diseases include (1) atelectasis-induced reductions in pulmonary compliance, (2) increased alveolar surface tension, and (3) stiffening of lung elastic tissue due to chronic disuse [12]. These factors may explain why some patients require higher inspiratory pressures, as suggested in previous reports [14]. However, our patient had no history of respiratory disease or atelectasis, and given the three-year progression with the declining lung capacity, a target of 40 cmH₂O was considered excessive. Instead, we applied a stepwise evaluation combining objective measures (expiratory volume) with subjective responses (subjective perception of lung expansion and post-inspiratory effects), ultimately determining 25 cmH₂O as the optimal setting. At this level, improvements in vocal projection and reduced breathlessness were observed without discomfort, supporting continued use after discharge.

Previous studies have reported improved respiratory compliance with relatively low inspiratory pressures in patients with restrictive ventilatory disorders. In ALS patients, Lechtzin et al. observed enhanced static compliance at pressures ranging from 19 to 30 cmH₂O, among individuals with %FVC between 35% and 105%, including three wheelchair users with values of 35%, 46%, and 59% [12]. Similarly, Sinha and Bergofsky demonstrated improved dynamic compliance in six kyphoscoliosis patients (%FVC 30%-58%, three with FVC <1.0 L) using intermittent positive pressure breathing at a mean inspiratory pressure of 22 cmH₂O [18]. These findings align with our case and further support the feasibility of achieving effective outcomes with relatively low inspiratory pressures in patients with severely reduced lung capacity. This underscores the importance of tailoring MI-E settings to optimize both efficacy and patient comfort.

In this case, MI-E was selected to perform LVR. Although various methods exist, including self-inflating resuscitation bags, LIC TRAINER2, and positive pressure ventilation, many require separate procurement and may impose additional costs. In contrast, MI-E is reimbursed under Japan’s healthcare system for ventilator-dependent neuromuscular patients, making it a practical choice for home care. A previous report [14] noted several advantages of MI-E: (1) no additional equipment required, (2) passive use without patient effort, (3) suitability for those with bulbar dysfunction, (4) minimal technical training required, and (5) reduced caregiver involvement aside from mask management. Despite these advantages, few studies have addressed how to individualize MI-E settings for LVR. Our method combined objective assessments with subjective feedback and required no special equipment. It can be readily implemented in diverse clinical environments. Once appropriately configured, MI-E supports sustained home use and reduces both financial and logistical burdens for patients and their families.

## Conclusions

This case demonstrates that an inspiratory pressure of 40 cmH₂O was not tolerated, while a lower, individually adjusted setting was acceptable and led to immediate functional benefits in an ALS patient undergoing LVR with MI-E. A stepwise adjustment based on both objective measures and patient-reported comfort led to improved vocal volume and reduced dyspnea. These findings underscore the value of a patient-centered approach over standardized pressure settings.
